# Age-related changes to triceps surae muscle-subtendon interaction dynamics during walking

**DOI:** 10.1038/s41598-021-00451-y

**Published:** 2021-10-28

**Authors:** William H. Clark, Jason R. Franz

**Affiliations:** Joint Department of Biomedical Engineering, University of North Carolina at Chapel Hill and North Carolina State University, 10206C Mary Ellen Jones Building, Chapel Hill, NC 27599 USA

**Keywords:** Ageing, Musculoskeletal system, Muscle, Tendons

## Abstract

Push-off intensity is largely governed by the forces generated by the triceps surae (TS) muscles (gastrocnemius-GAS, soleus-SOL). During walking, the TS muscles undergo different fascicle kinematics and contribute differently to biomechanical subtasks. These differences may be facilitated by the Achilles tendon (AT), which is comprised of subtendons that originate from the TS muscles. We and others have revealed non-uniform displacement patterns within the AT—evidence for sliding between subtendons that may facilitate independent muscle actuation. However, in older adults, we have observed more uniform AT tissue displacements that correlate with reduced push-off intensity. Here, we employed dual-probe ultrasound imaging to investigate TS muscle length change heterogeneity (GAS–SOL) as a determinant of reduced push-off intensity in older adults. Compared to young, older adults walked with more uniform AT tissue displacements and reduced TS muscle length change heterogeneity. These muscle-level differences appeared to negatively impact push-off intensity—evidenced by between-group differences in the extent to which TS muscle length change heterogeneity correlates with mechanical output across walking tasks. Our findings suggest that the capacity for sliding between subtendons may facilitate independent TS muscle actuation in young adults but may restrict that actuation in older adults, likely contributing to reduced push-off intensity.

## Introduction

Mechanical output at the ankle during push-off is an important determinant of walking performance and is significantly reduced with advancing age^1, 2^. More specifically, net ankle joint moment and mechanical power (i.e., push-off intensity) is largely governed by the forces generated by the lateral and medial gastrocnemius (GAS) and soleus (SOL) muscles that make up the triceps surae (TS)^3, 4^. Despite collectively transferring their force through a common distal tendon, the TS muscles undergo different magnitudes of fascicle length change during constant-velocity walking and, biomechanically, contribute differently to forward propulsion (primarily from GAS) and vertical support (primarily from SOL)^5–8^. These muscle-level differences may be facilitated by the architectural complexity of the Achilles tendon (AT), which itself is comprised of three distinct bundles of tendon fascicles, known as “subtendons”, that originate from GAS and SOL muscles^9–13^. Comparative work and our own in vivo evidence suggest that sliding between adjacent subtendons has the potential to allow differences in GAS vs. SOL muscle length change (i.e., TS muscle length change heterogeneity)^14–16^. Unfortunately, animal models of the aging tendon present with a proliferation of collagen cross-linking and prominent reductions in sliding between subtendons^15, 17^. In agreement with those findings, we have observed more uniform AT tissue displacement patterns in older adults that have the potential to disrupt muscle contractile independence^18^. These results, at least at the tendon level, appear to be clinically meaningful; during walking, more uniform subtendon displacements within the human AT correlate with reduced push-off intensity in older adults^19^. These observations allude to a fundamental change in the interaction between TS muscles and the AT as a determinant for reduced mechanical output—a finding that currently lacks direct empirical data during walking and has far reaching implications including the design and control of wearable assistive devices that attempt to overcome age-related deficits of forward propulsion.

The presence of subtendon sliding is mediated by the interfascicular matrix of the AT and has been commonly observed in rat and equine tendons (e.g.^14, 15^). In young adult humans, evidence for sliding is generally attributed to observations of differential tissue displacements at different depths of the AT (i.e., significant differences in tissue displacements attributed to the GAS and SOL subtendons) and has been shown during passive ankle rotation^20^, eccentric loading^21, 22^, and walking^23^. Studies in humans using advanced musculoskeletal modeling^24^ and studies in rats using electrical stimulation^25, 26^ suggest non-uniform tissue displacements are likely a result of differential force transmission from the TS muscles. Recently, to empirically characterize the origins of non-uniform tissue displacement patterns in the human AT, we introduced a dual-probe ultrasound imaging approach that enables simultaneous assessment of GAS vs. SOL muscle length change and tissue displacements in their associated regions of the AT^16^. Using this approach during fixed-end contractions, we found that differences between GAS and SOL muscle shortening (i.e., TS muscle length change heterogeneity) gave rise to anatomically consistent differences in subtendon tissue displacements^16^. As a logical extension of our dual-probe imaging work in younger adults, we more recently observed that more uniform AT tissue displacements in older adults during fixed-end contractions were accompanied by reduced TS muscle length change heterogeneity^18^.

TS muscle dynamics can precipitate anatomically consistent AT tissue displacement patterns (i.e., greater SOL shortening resulting in greater SOL subtendon displacement). Conversely, would an age-related reduction in the capacity for sliding between adjacent subtendons negatively influence TS muscle performance during walking? Although the answer is unclear, any change therein would likely influence the TS muscles’ relative contribution to forward propulsion^27^. In young adults, the majority of empirical studies suggest the gastrocnemius muscles are primarily responsible for governing changes in forward propulsion while the SOL is primarily responsible for governing changes in vertical support (e.g.^5–8^, though see^28, 29^ for alternative theories). However, experimental manipulations of walking speed affects both walking subtasks, thus confounding our interpretations in the specific context of forward propulsion^5, 30^. Fortunately, the application of horizontal aiding and impeding forces at fixed speeds has provided a more direct means to manipulate the mechanical demand for forward propulsion^6, 31^. Using horizontal forces in young adults, we recently revealed that compared to walking normally, increased mechanical demand for forward propulsion elicits larger peak GAS fascicle shortening than that of the SOL^8^. Therefore, it is likely that any disruption of GAS fascicle length via a reduction in the capacity for sliding between adjacent subtendons could deleteriously impact contributions to forward propulsion.

The purpose of this study was to investigate the magnitude of TS muscle length change heterogeneity (i.e., differences in GAS vs. SOL muscle length change) as a determinant of previously observed correlations between more uniform AT tissue displacements and reduced ankle joint mechanical output in older adults. To accomplish this, we used dual-probe dynamic ultrasound imaging during conditions that systematically altered the mechanical demand for forward propulsion via changes in speed and the application of horizontal aiding and impeding forces. First, we hypothesized that, compared to young adults, older adults would have (i) more uniform AT tissue displacements during the stance phase of walking that (ii) would be accompanied by smaller TS muscle length change heterogeneity. Second, we hypothesized that the magnitude of TS muscle length change heterogeneity would correlate with net ankle joint moment, power, and work performed during push-off. Finally, based on previous studies that suggest GAS muscles are more responsible than the SOL in governing changes in forward propulsion, we tested the secondary hypotheses that increases in the demand for forward propulsion would elicit larger TS muscle length change heterogeneity and AT non-uniformity in young, but not older adults. We based our secondary hypothesis on the premise that a larger capacity for sliding between adjacent Achilles subtendons may facilitate independent GAS vs. SOL muscle actuation and thus enhance biomechanical function during walking.

## Results

**Figure 1:**
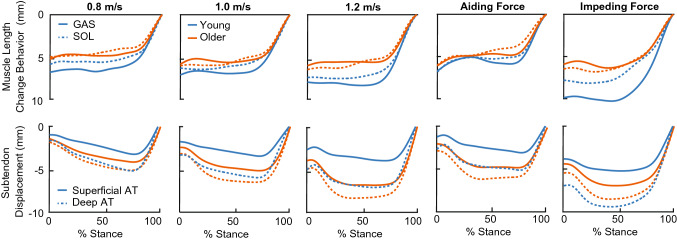
Time normalized, group average profiles for medial gastrocnemius (GAS) and soleus (SOL) longitudinal muscle length change (above) and superficial and deep Achilles tendon displacement (below) relative to toe-off for young (blue) and older (orange) adults.

Average ± standard deviation results and age-related post hoc statistical comparisons for each outcome measure are summarized in the Supplementary Information. Average preferred overground walking speed was not significantly different between young (1.3 ± 0.1 m/s) and older (1.2 ± 0.2 m/s) adults (P = 0.185, *g* = 0.586). Ankle range of motion increased with increased speed (P = 0.016, ${\eta }_{p}^{2}$ = 0.244) and horizontal force (P < 0.001, ${\eta }_{p}^{2}$ = 0.518). Compared to young, older adults walked with 4.7 ± 1.2° less ankle joint range of motion across our study protocol (Speed: P = 0.002, ${\eta }_{p}^{2}$ = 0.447; Force: P = 0.002, ${\eta }_{p}^{2}$ = 0.468). Here, we focus on the main effects of speed, horizontal forces, and age. We report age × speed or age × horizontal force interaction effects when significant. Group average muscle and subtendon profiles are shown in Fig. 1.

### Achilles tendon non-uniformity

**Figure 2:**
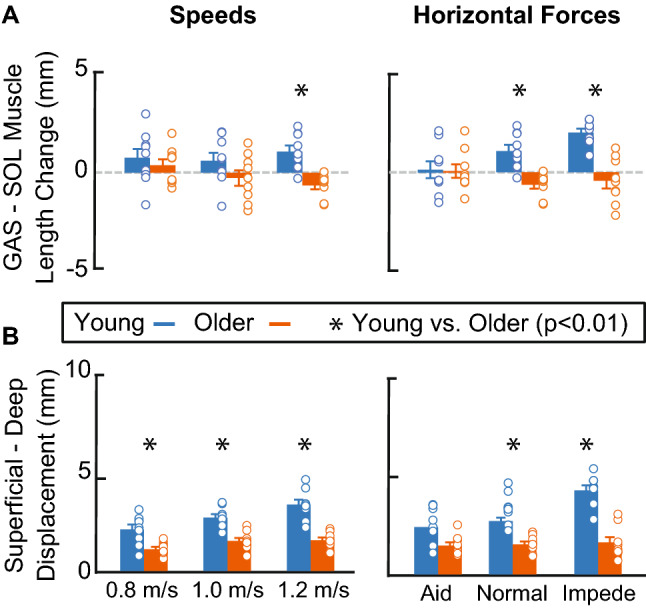
(**A**) Bar plots representing group average peak triceps surae muscle length change heterogeneity (i.e., medial gastrocnemius (GAS)–soleus (SOL) longitudinal muscle length change relative to toe-off). (**B**) Bar plots representing peak Achilles tendon non-uniformity (i.e., superficial—deep subtendon tissue displacements relative to toe-off). Single asterisks (*) represent significant differences between young (blue) and older (orange) adults (P < 0.01). Open circles represent individual data points. Error bars represent standard error.

Peak Achilles tendon (AT) non-uniformity (i.e., superficial—deep subtendon displacement) increased with increasing speed (P < 0.001, ${\eta }_{p}^{2}$ = 0.540) and horizontal force (P = 0.001, ${\eta }_{p}^{2}$ = 0.373, Fig. 2). Moreover, older adult peak AT non-uniformity was, on average across our study protocol, 49 ± 9% smaller than those in young (Speed: P < 0.001, ${\eta }_{p}^{2}$ = 0.721; Force: P < 0.001, ${\eta }_{p}^{2}$ = 0.820). Compared to that in young adults, significant interactions revealed that older adult peak AT non-uniformity was less sensitive to changes in speed (age × speed, P = 0.020, ${\eta }_{p}^{2}$ = 0.218) and horizontal forces (age × horizontal force, P = 0.004, ${\eta }_{p}^{2}$ = 0.294).

### Triceps surae muscle length change heterogeneity

**Figure 3:**
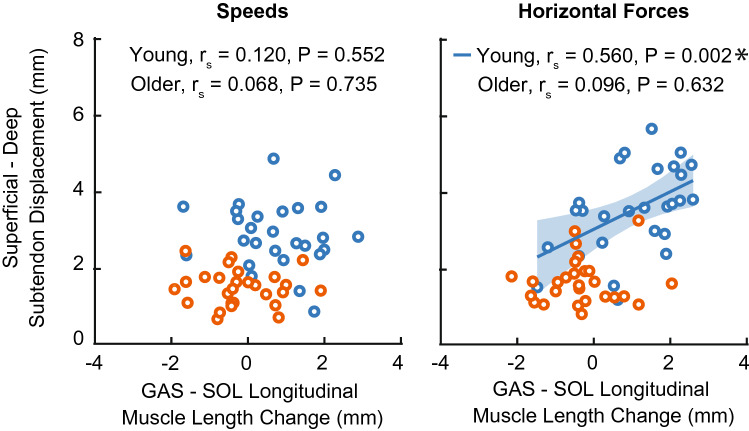
Spearman’s correlations between Achilles tendon non-uniformity (superficial—deep subtendon tissue displacements) and triceps surae muscle length change heterogeneity (i.e., medial gastrocnemius (GAS)–soleus (SOL) longitudinal muscle length changes) during conditions that alter speed (left) and horizontal force (right). Individual data points for young and older adults represented by blue and orange open circles, respectively. Single asterisks (*) represent significant correlations (P < 0.01).

At the instant of peak AT non-uniformity, TS muscle length change heterogeneity (i.e., GAS–SOL differences in longitudinal muscle length change) was unaffected by changes in speed (P = 0.325, ${\upeta }_{\mathrm{p}}^{2}$ = 0.068) and horizontal force (P = 0.096, ${\upeta }_{\mathrm{p}}^{2}$ = 0.136). Older adult TS muscle length change heterogeneity was, on average across our protocol, 18% smaller than those in younger adults (Speed: P < 0.025, ${\eta }_{p}^{2}$ = 0.275; Force: P < 0.001, ${\eta }_{p}^{2}$ = 0.630, Fig. 2), but with high variability (e.g., 41% smaller during impeding force condition and 4% larger during the aiding force condition). Moreover, compared to those in young adults, significant interactions revealed that older adult TS muscle length change heterogeneity was less sensitive to changes horizontal forces (age × horizontal force, P = 0.003, ${\eta }_{p}^{2}$ = 0.310). We did not observe a significant correlation between TS muscle length change heterogeneity and AT non-uniformity during conditions that altered speed for young or older adults (P-values ≥ 0.552). However, TS muscle length change heterogeneity positively correlated with AT non-uniformity in young (P = 0.002, r_s_ = 0.560, 95% CI [0.217, 0.780]) but not older adults (P = 0.632) during conditions that altered horizontal force (Fig. 3).

### Ankle joint mechanical output

Peak ankle moment, peak ankle power, and positive ankle push-off work increased with increasing speed (P-values < 0.001, ${\eta }_{p}^{2}$ ≥ 0.657) and horizontal force (P-values < 0.001, ${\eta }_{p}^{2}$ ≥ 0.362). Although we did not observe a significant main effect of age on peak ankle moment (Speed: P = 0.462, ${\eta }_{p}^{2}$ = 0.034; Force: P = 0.216, ${\eta }_{p}^{2}$ = 0.094) or positive push-off work (Speed: P = 0.104, ${\eta }_{p}^{2}$ = 0.156; Force: P = 0.063, ${\eta }_{p}^{2}$ = 0.200), older adults did walk with significantly smaller peak ankle power than young during conditions that altered horizontal force (Speed: P = 0.079, ${\eta }_{p}^{2}$ = 0.181; Force: P = 0.040, ${\eta }_{p}^{2}$ = 0.238). Ankle moment and ankle power profiles are reported in the Supplementary Information.

**Figure 4:**
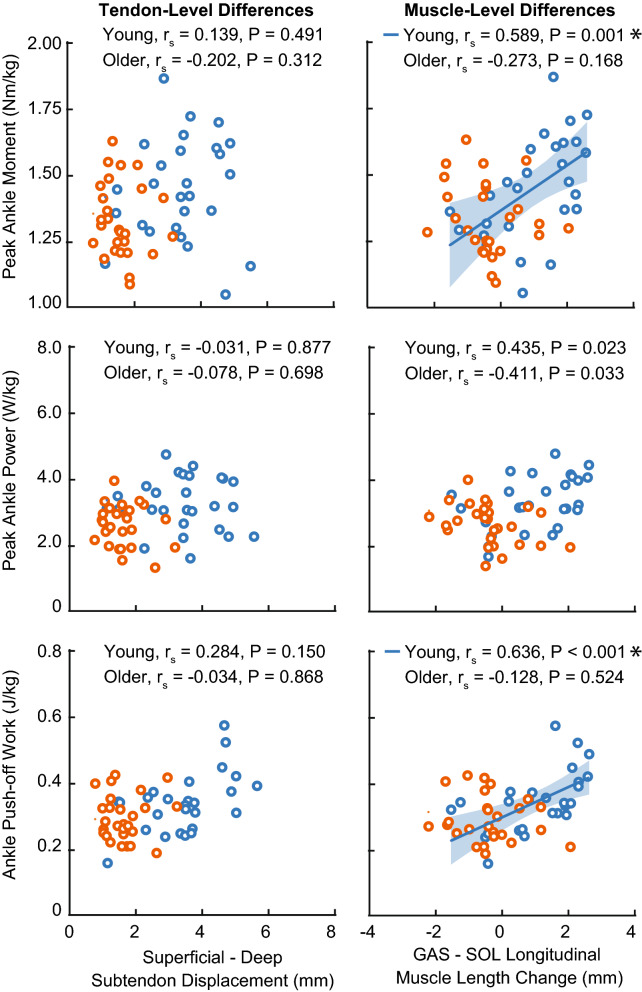
Spearman’s correlations between push-off intensity (i.e., peak ankle moment, peak ankle power, and ankle push-off work) and (left) Achilles tendon non-uniformity (superficial—deep subtendon tissue displacement) and (right) triceps surae muscle length change heterogeneity (medial gastrocnemius (GAS)–soleus (SOL) longitudinal muscle length change) during conditions that alter the mechanical demand for forward propulsion via horizontal forces. Individual data points for young and older adults represented by blue and orange open circles, respectively. Single asterisks (*) represent significant correlations (P < 0.01).

We did not observe a significant correlation between AT non-uniformity and ankle joint kinetics for any condition or age group (Fig. 4). For conditions that altered speed, we only observed a significant correlation between young adult TS muscle length change heterogeneity and peak ankle moment (P = 0.004, r_s_ = 0.535, 95% CI [0.183, 0.765]). During conditions that altered horizontal force, TS muscle length change heterogeneity positively correlated with peak ankle moment (P = 0.001, r_s_ = 0.589, 95% CI [0.258, 0.796]) and positive ankle push-off work in young (P < 0.001, r_s_ = 0.636, 95% CI [0.326, 0.822]), but not peak ankle power (P = 0.023). In older adults, all correlations were non-significant (peak ankle moment: P = 0.168; peak ankle power: P = 0.033; positive ankle push-off work: P = 0.524).

### Superficial and deep subtendons

Peak superficial and deep subtendon tissue displacements increased with increasing speed (P-values < 0.001, ${\upeta }_{\mathrm{p}}^{2}$ ≥ 0.454) and horizontal force (P-values ≤ 0.001, ${\upeta }_{\mathrm{p}}^{2}$ ≥ 0.443). Peak superficial subtendon tissue displacements in older adults were significantly larger than in young adults (Speed: P = 0.001, ${\eta }_{p}^{2}$ = 0.538; Force: P = 0.001, ${\eta }_{p}^{2}$ = 0.495). Conversely, we did not observe a significant main effect of age on peak deep subtendon tissue displacements (Speed: P = 0.206, ${\eta }_{p}^{2}$ = 0.098; Force: P = 0.412, ${\eta }_{p}^{2}$ = 0.043).

### GAS and SOL muscles

At the instant of peak AT non-uniformity, GAS and SOL longitudinal muscle length change relative to toe-off increased with increasing speed (P-values ≤ 0.009, ${\upeta }_{\mathrm{p}}^{2}$ > 0.257) and horizontal force (P-values < 0.001, ${\upeta }_{\mathrm{p}}^{2}$ ≥ 0.556). Older adult GAS longitudinal muscle length changes were significantly smaller than those in young adults (Speed: P = 0.011, ${\eta }_{p}^{2}$ = 0.343; Force: P < 0.001, ${\eta }_{p}^{2}$ = 0.537). Moreover, compared to those in young adults, significant interactions revealed that older adult GAS longitudinal muscle length changes were less sensitive to changes in horizontal force (age × horizontal force, P < 0.001, ${\eta }_{p}^{2}$ = 0.520). Conversely, we did not observe a significant main effect of age on SOL longitudinal muscle length change (Speed: P = 0.240, ${\eta }_{p}^{2}$ = 0.085; Force: P = 0.099, ${\eta }_{p}^{2}$ = 0.161). At the instant of peak AT non-uniformity, both GAS and SOL muscle–tendon unit length change relative to toe-off increased with increasing speed (P-values < 0.001, ${\upeta }_{\mathrm{p}}^{2}$ > 0.658). However, only SOL muscle–tendon unit length changes increased with horizontal force (P = 0.003, ${\eta }_{p}^{2}$ = 0.298); GAS muscle–tendon unit length changes were unaffected (P = 0.560, ${\eta }_{p}^{2}$ = 0.036). Both older adult GAS and SOL muscle–tendon unit length changes were significantly smaller than those in young adults (Speed: P-values ≤ 0.001, ${\eta }_{p}^{2}$ ≥ 0.500; Force: P-values ≤ 0.002, ${\eta }_{p}^{2}$ ≥ 0.459). Results for GAS and SOL average fascicle operating length and peak shortening velocity are reported in the Supplementary Information.

## Discussion

In this study, we investigated the magnitude of triceps surae (TS) muscle length change heterogeneity (i.e., differences in GAS vs. SOL muscle length change) as a determinant of previously observed correlations between more uniform Achilles tendon (AT) tissue displacements and reduced ankle joint mechanical output in older adults. We used dual-probe ultrasound imaging to simultaneously quantify muscle length change and subtendon tissue displacements during conditions that systematically altered the mechanical demand for forward propulsion via changes in speed and the application of horizontal aiding and impeding forces. In support of our hypotheses, older adults walked with a significant reduction in AT non-uniformity and reduced TS muscle length change heterogeneity. Moreover, we observed between-group differences in the extent to which TS muscle length change heterogeneity positively correlates with push-off intensity (i.e., moment, power, and positive push-off work) across a range of tasks during walking. As we elaborate in more detail below, these findings suggest that age-related changes to the interaction between TS muscles and the AT negatively affect ankle joint mechanical output.

In our study, despite walking with identical experimental manipulations, older adults exhibited hallmark deficits in push-off intensity that were evident via conventional biomechanical analysis of joint-level kinematics and kinetics. Here, in agreement with the prevailing literature, older adults walked with a characteristic reduction in ankle joint range of motion^32^. Moreover, in response to increased mechanical demand for forward propulsion, older adults walked with significantly smaller peak ankle power than young adults (e.g., − 24% with impeding forces). However, contrary to several prior studies, we did not observe an effect of age on peak ankle moment or positive ankle push-off work during walking, likely as a result of our relatively slow range of walking speeds^33^. Yet, similar to our findings, Knaus et al. revealed that older adults who walked with similar ankle moment generation to younger adults presented with deleterious changes in AT structure–function relations that are not measurable using conventional biomechanical analyses alone^34^.

Our results for AT tissue displacements agree well with previous findings in terms of age-related differences and changes in response to increased mechanical demand. Our research group previously revealed that the magnitude of AT non-uniformity increased with faster speeds and, when pooling young and older adults together, positively correlated with push-off intensity (non-uniformity vs. moment: R = 0.63; vs. power: R = 0.39; vs. positive work: R = 0.44; P-values < 0.01)^19^. Moreover, this prior work showed more uniform AT tissue displacements in older adults (age-related differences up to 41% at 1.25 m/s), consistent with animal models of aging tendon and a reduced capacity for sliding^15, 17^. Here, we report similar age-related reductions in AT non-uniformity that increased at faster walking speeds (up to 53% smaller in older adults at 1.20 m/s). We also add that those age-related differences in AT non-uniformity were even larger in response to impeding forces designed to increase the mechanical demands for propulsion (age-related differences up to 63% during the impeding force condition).

Unlike our previous work which pooled young and older adults together, here we separated the age groups to better elucidate changes in the interaction between muscle and tendon and the resultant implications on ankle joint mechanical output. In young adults, across changes in horizontal force, the magnitude of AT non-uniformity positively correlated with larger differences in TS muscle length change heterogeneity (i.e., GAS vs. SOL muscle length change). However, these correlations were absent in older adults. Consistent with our previous results during fixed-end contractions^18^, more uniform AT tissue displacements in older adults coincided with a reduction in TS muscle length change heterogeneity. Moreover, we add that older adult TS muscle length change heterogeneity and AT non-uniformity were significantly less sensitive to changes in horizontal force. We interpret these cumulative findings to suggest that the AT may facilitate independent TS muscle actuation in young adults and, likely due to an age-related decrease in the capacity for sliding between adjacent subtendons, may restrict that actuation in older adults. Indeed, compared to younger tendons, older tendons present with a proliferation of interfascicle adhesions which may underly prominent reductions in the capacity for sliding between adjacent subtendons^15, 17^. If more uniform AT tissue displacements restrict TS muscle length change heterogeneity, our results suggest this primarily affects the GAS muscle-subtendon unit. In response to horizontal forces, GAS muscle length change and superficial subtendon displacement (i.e., tissue associated with the GAS) was significantly different in young versus older adults, while SOL muscle-subtendon behavior remained unchanged. Moreover, older adult GAS operating lengths were shorter than those of young adults, while SOL operating lengths were preserved (see Supplementary Information).

The mechanisms underlying age-related deficits in push-off intensity appear to be strongly influenced by TS muscle-AT interaction. In young adults, the magnitude of TS muscle length change heterogeneity positively correlated with measures of push-off intensity (i.e., peak ankle moment and positive push-off work). In our view, these findings provide a mechanistic link for previously observed correlations between the magnitude of AT tissue non-uniformity and ankle joint mechanical output. Consistent with our overarching hypothesis that more uniform AT tissue displacements disrupts TS contractile dynamics and thus ankle joint mechanical output, we did not observe any significant positive correlations between TS muscle length change heterogeneity and push-off intensity in older adults. Recently, we combined electromyography, ultrasound imaging, and musculoskeletal modeling in the same subjects and revealed that the biarticular gastrocnemius muscles play a more significant role than the uniarticular SOL in governing changes in forward propulsion in young adults^8^. It follows that any disruption in muscle contractile dynamics would deleteriously impact their relative contribution to forward propulsion, thereby affecting push-off intensity. Indeed, length is a critical determinant of muscle force production, and by extension, moment and power generation^35^. However, it is important to note that conclusions based on muscle lengths alone are fundamentally incomplete as they relate to muscle–tendon force estimates^36^. For example, the stretch and recoil of the AT can produce substantial forces during the push-off phase of walking and likely governs the speed of ankle rotation as energy is returned, thus significantly impacting ankle moment and power generation^37^.

Our results may have important implications for the design and control of wearable assistive devices that attempt to overcome age-related deficits of forward propulsion. Our study suggests more uniform AT tissue behavior may primarily affect the GAS muscle-subtendon unit. To date, the majority of studies disproportionately target the uniarticular SOL through the design and prescription of ankle exoskeletons (e.g.^38, 39^). At least one study has shown that exoskeleton configurations that mimic the biarticular GAS yield larger reductions in metabolic cost during walking than those that mimic the uniarticular SOL^40^. Finally, although additional work is certainly warranted, our results may have implications on therapeutic techniques (e.g., hyaluronic acid injections^41^), rehabilitation strategies (e.g., preferentially strengthening the GAS), or surgical techniques that seek to maintain or restore tendon function due to injury or the aging process^42^.

Cumulatively, our results suggest that advancing age deleteriously impacts TS muscle-AT interaction, thereby reducing ankle joint mechanical output. However, there are several factors that may confound our mechanistic understanding of muscle–tendon interaction and its role in governing age-related reductions in peak ankle moment and power output. For example, age-related changes to neural drive may result in older adults utilizing a different neural control strategy than young adults to meet the demand for greater push-off intensity. Here, we observed a significant age × horizontal force interaction effect on both AT non-uniformity and TS muscle length change heterogeneity. Contrary to that of the GAS muscle–tendon unit, we did not observe a significant effect of age on SOL muscle length change, SOL subtendon displacement, SOL average fascicle operating length, or SOL peak shortening velocity. The preservation of SOL muscle–tendon unit behavior likely suggests that older adults have a greater relative reliance on SOL muscle output compared to younger adults. Type II fiber physiological cross-sectional area (PSCA), representing approximately 43% of total PCSA in the GAS, is reduced by age more than that of Type I fibers, representing approximately 80% of total PCSA in the SOL^43^. Moreover, shorter GAS average fascicle operating lengths in older adults suggests a leftward shift on the force–length curve and smaller force generating capacity compared to young adults. The resultant inability to modulate force to meet task demands could further increase reliance on the SOL. However, not all literature suggests older adults have a greater reliance on the SOL. Schmitz et al. observed greater age-related decreases in SOL muscle activation than MG activation and concluded that the SOL diminishes its contribution to forward propulsion with age^44^. Disparate changes in the biarticular GAS vs uniarticular SOL muscle activation may arise from an attempt to stabilize the ankle (affecting both GAS and SOL) and knee (affecting GAS only)^45^.

We cannot exclude the possibility that joint posture contributes in part to age-related differences in muscle-subtendon interaction dynamics. In our study, a post-hoc analysis revealed no significant effect of age on knee range of motion (Speed: P = 0.236, ${\eta }_{p}^{2}$ = 0.087; Force: P = 0.580, ${\eta }_{p}^{2}$ = 0.020) or peak knee flexion power (Speed: P = 0.082, ${\eta }_{p}^{2}$ = 0.177; Force: P = 0.075, ${\eta }_{p}^{2}$ = 0.185). On the other hand, older adults walked with a significant reduction in ankle range of motion. Accordingly, GAS and SOL muscle–tendon unit lengths changes were significantly smaller in older adults than in younger adults. This reduction alludes to less overall muscle–tendon unit strain and could itself diminish TS muscle length change heterogeneity, AT tissue non-uniformity, and push-off intensity in older adults. However, our findings are fully consistent with those we have previously revealed during isolated contractions, where MTU lengths and joint postures were prescribed^18^. Our results also contradict those we would anticipate from joint posture effects alone; namely, the larger GAS subtendon tissue displacements in older adults and the indistinguishable SOL muscle length changes between age groups. Continued mechanistic work in this area should focus on untangling the relative contributions of different factors governing age-related changes to muscle-subtendon interaction dynamics during walking.

Here, we did not take measurements of muscle strength. However, it is possible that decreased TS muscle force generation in older adults, even if homogenous across the TS muscles, could diminish non-uniform AT tissue displacements. Yet, resistance training programs designed to increase TS muscle strength have failed to directly translate to greater measures of ankle moment or walking speed^46–48^. It is unclear what effect training would have on AT non-uniformity or TS muscle length change heterogeneity. In addition to TS muscle strength, age-related changes in AT tissue properties may explain the disparate directionality in the relation between ankle power and the magnitude of TS muscle length change heterogeneity in young vs. older adults. As one example, the GAS and SOL subtendons likely differ in stiffness^25^. As such, any age-related changes in subtendon tissue compliance may unevenly diminish force transmission from the TS muscles^49^.

There are several important limitations to this study. First, this study uses a generalized approximation of TS muscle-AT anatomy that may not be fully captured using two-dimensional ultrasound imaging. For example, we only imaged the posteromedial SOL, which may differ from the anterior, posterior, medial, and lateral components^50^. Second, we have previously outlined limitations in our muscle^8^ and tendon tracking techniques^19^. For muscle, we attempted to mitigate errors in automated tracking by recording at a higher frame rate (76 fps) and performing frame-by-frame manual corrections of automated results^51^. All fascicle length and pennation angle intraclass correlation coefficients (ICC) were above 0.50. Young (older) adult ICC values averaged 0.87 ± 0.1 (0.88 ± 0.1) and 0.84 ± 0.1 (0.87 ± 0.1) for GAS and SOL fascicle lengths, respectively. Young (older) adult ICC values averaged 0.71 ± 0.1 (0.73 ± 0.1) and 0.68 ± 0.1 (0.73 ± 0.1) for GAS and SOL pennation angles, respectively. For tendon, we attempted to mitigate motion artifacts by limiting our maximum walking speed to 1.2 m/s. Third, we only report subtendon tissue displacements, an outcome we can measure with a higher level of confidence than subtendon elongation. Estimating subtendon tissue elongation relative to the tendon insertion point on the calcaneus can be prone to errors^52^. Fourth, we made no attempt to estimate forces transmitted from individual muscles through individual subtendons, which are likely heterogeneous and highly complex^53^. Finally, the majority of our conclusions are based on correlations that cannot definitively convey causal links.

In this study, we provide in vivo evidence that older adults walk with a significant reduction in AT non-uniformity and reduced TS muscle length change heterogeneity (i.e., differences between medial gastrocnemius and soleus longitudinal muscle length change). Moreover, we observed between-group differences in the extent to which TS muscle length change heterogeneity positively correlates with push-off intensity (i.e., moment, power, and positive push-off work) across a range of tasks during walking. Overall, we interpret our findings to suggest that a greater capacity for sliding between adjacent subtendons may facilitate independent triceps surae muscle actuation in young adults but a reduced capacity for sliding may restrict that actuation in older adults. The resultant disruption in TS muscle contractile dynamics likely contributes at least in part to hallmark reductions in push-off intensity during walking in older adults.

## Methods

### Subjects and protocol

We recruited healthy young adults between 18 and 35 years of age and healthy older adults between 65 and 80 years of age. We screened and excluded subjects who reported a lower extremity injury in the last 6 months, used a leg prosthesis, needed an assistive walking device, or were taking medication that causes dizziness. We report data for 9 young (24 ± 4 years, 74.3 ± 9.9 kg, 1.8 ± 0.1 m, 5M/4F) and 9 older (74 ± 4 years, 66.8 ± 5.9 kg, 1.7 ± 0.1 m, 6M/3F) adults. Prior to participation, all subjects provided written informed consent. All methods were approved by the University of North Carolina at Chapel Hill Biomedical Sciences Institutional Review Board (16-0379) and were carried out in accordance with relevant guidelines.

**Figure 5:**
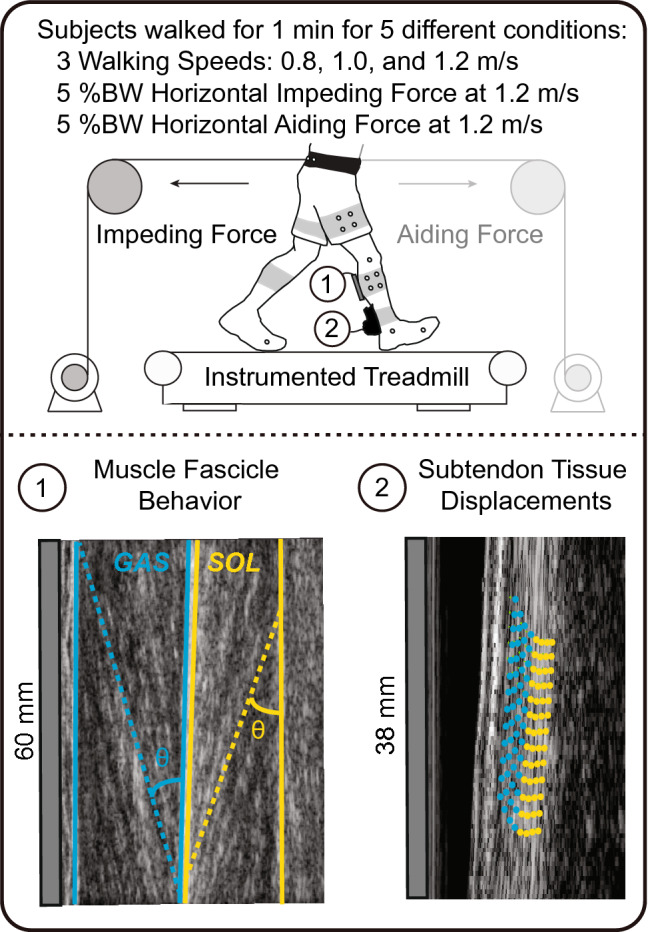
Schematic showing experimental manipulations and the application of dual-probe ultrasound imaging of (1) medial gastrocnemius (GAS-blue) and soleus (SOL-yellow) muscle contractile behavior and (2) Achilles subtendon tissue displacements. Figure in upper panel created using Adobe Illustrator (version 25.4.1, available from: https://adobe.com/products/illustrator).

Before data collection, we determined each subject’s preferred walking speed as the average of three times taken to walk the middle 2 m of a 10 m walkway. Subjects then walked on an instrumented treadmill (Bertec Corp., Columbus, Ohio, USA) for 6 min at their preferred walking speed (above 1.0 m/s) to precondition their triceps surae (TS) muscles and Achilles tendon (AT) units and acclimate to treadmill walking^54^. Subjects then walked for 1 min each at a range of speeds (0.8, 1.0, and 1.2 m/s) and again at 1.2 m/s (“Normal”) with: (i) a 5% body weight horizontal aiding force and (ii) a 5% body weight horizontal impeding force, as shown in Fig. 5. A feedback-controlled, motor-driven horizontal force system applied horizontal aiding and impeding forces, as described previously^55^. Briefly, in real-time, a custom LabVIEW interface (cRIO-9064, National Instruments, Austin, TX, USA) controlled a servo motor (Kollmorgen, Radford, VA, USA) in series with a horizontal cable attached to a waist belt worn by the subject. During trials with horizontal impeding or aiding forces, we consistently encouraged subjects to maintain upright posture (i.e., avoid excess trunk leaning). We fully randomized by walking condition and provided 2 min of rest between conditions. Subjects were barefoot throughout all walking trials to facilitate proper placement of ultrasound transducers. We collected all ensuing data after subjects reported being comfortable walking at the targeted speed.

### Experimental measurements and analyses

For all trials, 12 cameras (Motion Analysis Corporation, Santa Rose, CA, USA) recorded the 3-D positions of retroreflective markers attached to each subject’s sacrum and bilateral anterior and posterior superior iliac spines, lateral femoral condyles, lateral malleoli, 1st and 5th metatarsal heads, and calcanei. To improve segment tracking, we secured right and left leg thigh and shank marker clusters. We filtered marker position and ground reaction force (GRF) data using a 4th order low-pass zero-phase Butterworth filter with cutoff frequencies of 6 Hz and 100 Hz, respectively. We scaled a 7-segment, 18 degree of freedom model of each subject’s pelvis and lower limbs using marker position data from a standing trial^56^ and then updated the model to include functional hip joint centers^57^. A global optimization technique^58^ calculated ankle joint moment, power, and positive ankle push-off work for each stride corresponding to ultrasound data as detailed in the following section.

### Ultrasound measurements

A 7 MHz, 60 mm ultrasound transducer (LV7.5/60/128Z-2, UAB Telemed, Vilnius, Lithuania) placed over the mid-belly of the right leg medial gastrocnemius (GAS) recorded cine B-mode images at 76 frames per second using an image depth of 50 mm. This transducer placement and depth enabled synchronized assessment of GAS and soleus (SOL) fascicle behavior in the same imaging plane. We secured the ultrasound probe using a custom 3-D printed probe holder made of a flexible filament (NinjaFlex, Fenner Inc., Manheim, PA, USA) and wrapped in Coban (3 M, St. Paul, MN, USA). Simultaneously, a 10 MHz, 38 mm ultrasound transducer (L14-5 W/38, Ultrasonix Corp., Richmond, BC) placed over the right leg free AT, approximately 6 cm proximal to the calcaneal insertion, recorded 128 lines of ultrasound radiofrequency data at 155 frames per second using an image depth of 20 mm. This transducer placement and depth enabled synchronized assessment of two equally sized tendon depths—superficial and deep—corresponding to subtendon tissue thought to arise from the GAS and SOL, respectively. This characterization represents the most prevalent anatomical arrangement in cadaveric studies^10, 13, 59, 60^. We secured the second ultrasound probe using a custom probe holder made of a layered plastazote foam and wrapped in Coban.

For each ultrasound transducer, a 1000 Hz binary analog synchronization signal indicated the start and stop of each ultrasound video using a wave form generator (SDG1025, SIGLENT, Shenzhen). We co-registered ultrasound data with GRF data and detected heel-strike and toe-off gait events using a 10 N vertical GRF threshold. Finally, we quantified time series of GAS and SOL fascicle behavior (i.e., length and pennation angle) and superficial and deep AT tissue behavior (i.e., subtendon displacements), from the same 2 strides per condition, as described in detail below. The same investigator processed all ultrasound data to minimize inter-investigator variability.

### Triceps surae muscle kinematics

At the first co-registered heel-strike, we defined a static polygon region of interest surrounding the GAS and SOL muscle bellies and corresponding superficial and deep aponeuroses. For each muscle, we defined one fascicle from superficial to deep aponeurosis that most represented the muscle belly. An open-source MATLAB routine, UltraTrack^61^, based on an affine extension to an optic flow algorithm, quantified time series GAS and SOL fascicle length and pennation angle. UltraTrack defines pennation angle relative to the horizontal axis of the image. Thus, to more accurately define pennation angle, for each muscle, we quantified the angle of the deep aponeurosis relative to the horizontal axis of the image and then subsequently applied a correction to fascicle pennation angle. For fascicle tracking and superficial aponeurosis pennation angle corrections, we visually confirmed the results and applied manual corrections when necessary. We filtered all manually corrected fascicle lengths and pennation angles using a 4th order low-pass zero-phase Butterworth filter with a cutoff frequency of 6 Hz and then averaged the results over 2 strides (heel-strike to heel-strike). For fascicle behavior, we report time series GAS and SOL average fascicle operating length and velocity (i.e., derivative of fascicle length with respect to time). For a more direct comparison of longitudinal tissue displacements, we multiplied muscle fascicle length by the cosine of the pennation angle to compute longitudinal muscle length along the AT line of action. Then, to place our results in the context of distal to proximal subtendon displacement as described below, we normalized muscle length change results by their length at toe-off. Finally, for muscle behavior, we report TS muscle length change heterogeneity, defined as the stance-phase peak differences in longitudinal muscle length change (i.e., GAS–SOL).

### Achilles subtendon tissue kinematics

A 2-D speckle tracking algorithm quantified localized Achilles subtendon tissue displacements using previously published techniques^62^. Although muscle fascicle data during gait are usually represented from heel-strike to heel-strike to fully characterize the stretch shortening cycle, 2-D elastography speckle tracking is more accurate when the initial frame corresponds to an instant of negligible tendon force (i.e., toe-off)^62, 63^. As such, at the first co-registered toe-off, we created a rectangular region of interest (~ 15 × 3 mm grid of nodes with 1 × 0.5 mm spacing, encompassing only AT tissue) on a reconstructed B-mode image using the raw radiofrequency data. A 2 × 1 mm kernel centered at each nodal position contained up-sampled (4×) radiofrequency data and acted as a search window for successive 2-D normalized cross-correlation functions. Second order polynomials then regularized frame-to-frame nodal displacements that maximized the 2-D cross correlations, using a threshold of r = 0.7^64^. In rare cases when correlations fell below threshold, we determined new displacements for the relative frames based on a cubic interpolation of neighboring, highly correlated frames, and in the subsequent frame we added the median-filtered (3 × 3 nodes) nodal displacement. For each stride (toe-off to subsequent toe-off), we averaged the forward and backward tracking results. The resultant cumulative displacements represented the longitudinal displacements of superficial (i.e., originating from the GAS) and deep (i.e., originating from the SOL) subtendon tissue. We filtered all subtendon tracking results using a 4th order low-pass zero-phase Butterworth filter with a cutoff frequency of 6 Hz and then averaged the results over 2 strides (toe-off to toe-off). Finally, we report AT non-uniformity as the stance-phase peak differences in longitudinal displacements (i.e., superficial—deep).

### Statistical analysis

For each subject and condition (i.e., 0.8, 1.0, 1.2 m/s, aiding, and impeding), we confirmed appropriate between-stride variability for fascicle length and pennation angle measurements using intraclass correlation coefficients (ICC)^65^. ICC values < 0.50 were considered poor, 0.50–0.75 were moderate, 0.75–90 were good, and > 0.90 were excellent^66^. Shapiro–Wilk tests confirmed normality appropriate for analyses of variance for each outcome measure. Mauchly’s test of sphericity assessed the variance of the differences for each outcome measure. When the assumption of normality was violated, Greenhouse–Geisser adjustments were applied. Mixed factorial ANOVAs tested for the effect of age (young vs. older adults) and speed (i.e., 0.8, 1.0, and 1.2 m/s) or horizontal pulling force (i.e., 1.2 m/s, aiding, and impeding) on ankle joint kinematics (i.e., range of motion) and kinetics (i.e., peak ankle moment, peak ankle power, and positive ankle push-off work during stance), GAS and SOL fascicle kinematics (i.e., peak fascicle shortening velocity, average fascicle length), peak AT non-uniformity during stance, and peak TS muscle length change heterogeneity at the instant of peak tendon non-uniformity. Mixed factorial ANOVAs used an alpha level of 0.05. When significant main effects were found, two-tailed independent samples *t* tests with Bonferroni corrections identified age-related differences. Additionally, two-tailed paired samples *t* tests with Bonferroni corrections identified differences between TS muscles (i.e., GAS vs. SOL) and Achilles subtendons (i.e., superficial vs. deep). Finally, for young and older adults, we calculated Bonferroni corrected Spearman’s correlation coefficients between peak AT non-uniformity, TS muscle length change heterogeneity, and ankle joint kinetic outcomes. Pairwise comparisons and Spearman’s correlations used an alpha level of 0.01. Finally, we report effect sizes as ${\eta }_{p}^{2}$ and Hedges’ *g* (with bias correction for sample size < 20) for main effects and pairwise comparisons, respectively. Values of *g* > 0.20, 0.50, and 0.80 indicated small, moderate, and large effects, respectively^67^.

## Supplementary Information

Supplementary Information 1.

Supplementary Information 2.

Supplementary Video 1.

Supplementary Video 2.

## Data Availability

The datasets generated during the current study are available from the corresponding author on request.
