# Supplementary material for: Age-related changes to triceps surae muscle-subtendon interaction dynamics during walking
Source: Sci Rep. 2021 Oct 28;11:21264. doi: 10.1038/s41598-021-00451-y (PMC8553842; doi:10.1038/s41598-021-00451-y)
Supplement: Supplementary file 1 — Supplementary Information 1. [file 41598_2021_451_MOESM1_ESM.pdf]

Age-Related Changes to Triceps Surae Muscle-Subtendon Interaction Dynamics During Walking

William H. Clark and Jason R. Franz*

Joint Department of Biomedical Engineering, University of North Carolina at Chapel Hill and North Carolina State University, Chapel Hill, NC, USA

*Author for correspondence

Jason R. Franz

Email: jrfranz@email.unc.edu

Phone: (919) 966-6119

Fax: (919) 966-2963

10206C Mary Ellen Jones Building

Chapel Hill, NC 27599

SUPPLEMENTARY INFORMATION

Fascicle Operating Length

As seen in Supplementary Figure S3, average GAS fascicle operating lengths decreased with increasing speed and horizontal forces (Speed: P = 0.034, η_p^2 = 0.191; Force: P < 0.001, η_p^2 = 0.501); SOL fascicle operating lengths only decreased in response to increases in horizontal force (Speed: P = 0.107, η_p^2 = 0.142; Force: P = 0.002, η_p^2 = 0.315). Older adults had significantly shorter average GAS fascicle operating lengths than young adults (Speed: P = 0.004, η_p^2 = 0.409; Force: P = 0.005, η_p^2 = 0.398). Moreover, significant interactions revealed older adult GAS fascicle operating lengths were less sensitive to changes in horizontal force than that of younger adults (age×horizontal force, P = 0.002, η_p^2 = 0.327). Conversely, we did not observe a significant main effect of age on average SOL fascicle operating length (Speed: P = 0.342, η_p^2 = 0.057; Force: P = 0.264, η_p^2 = 0.077).

Muscle Fascicle Velocity

The magnitudes of peak GAS and SOL fascicle shortening velocity increased with increasing speed (P-values ≤ 0.024, $\eta_p^2 \geq$ 0.209) but were unaffected by horizontal forces (P-values ≥ 0.123, $\eta_p^2 \leq$ 0.123). We did not observe a significant main effect of age on the magnitudes of peak GAS or SOL fascicle shortening velocity during conditions that altered speed (P-values ≥ 0.110, $\eta_p^2 \leq$ 0.152). However, older adult GAS fascicle shortening velocity was significantly slower than young adults during conditions that altered horizontal force (P = 0.017, η_p^2 = 0.306); SOL fascicle shortening velocity was unaffected by age (P = 0.124, η_p^2 = 0.141). We have previously proposed that a shift to slower fascicle shortening velocities in older adults may serve to meet the muscle force-generating demands of walking in the presence of muscle weakness[54].

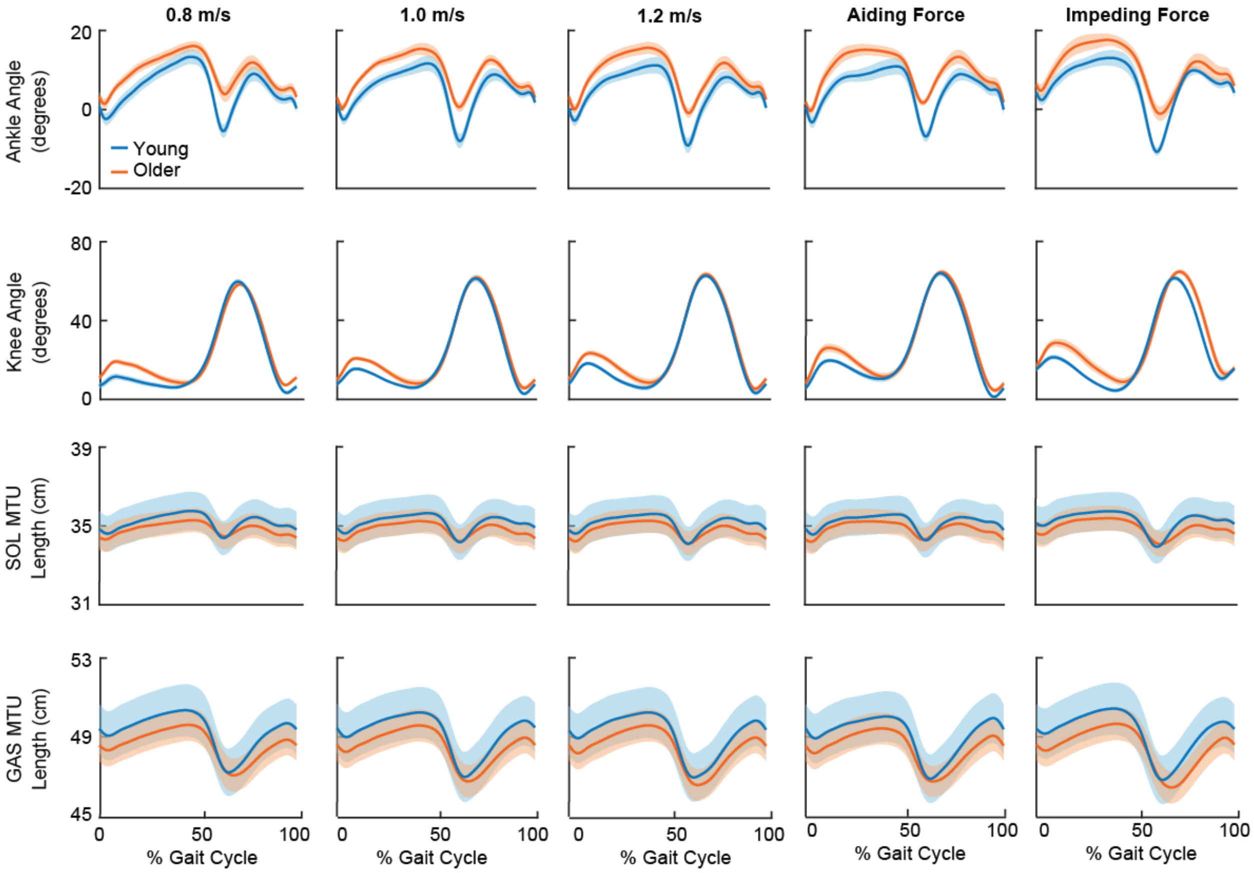

Supplementary Figure S1. Group average ankle angle, knee angle, soleus (SOL), and medial gastrocnemius (GAS) muscle-tendon unit (MTU) length for young (blue) and older (orange) adults during conditions that altered speed (left) and horizontal force (right).

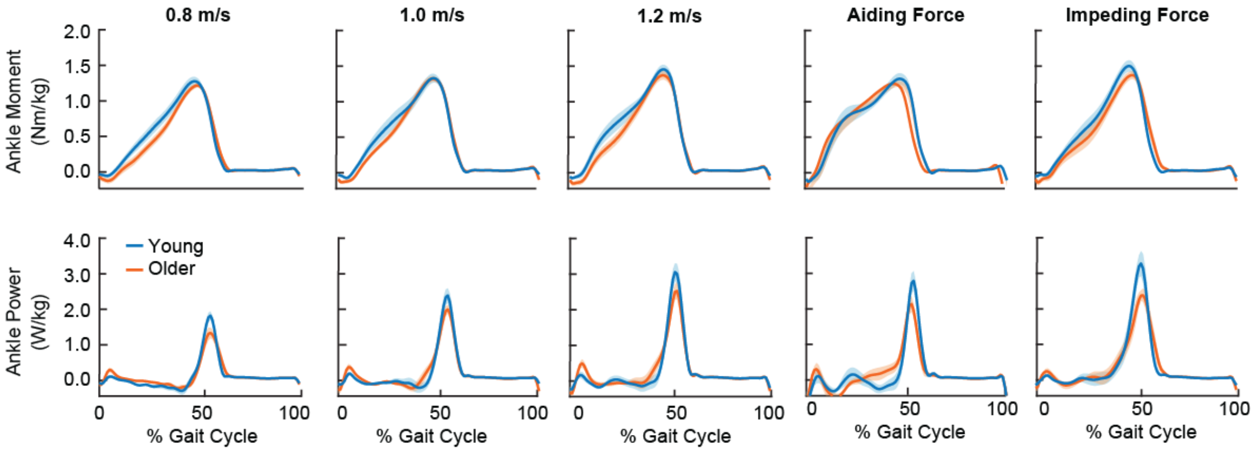

Supplementary Figure S2. Group average ankle moment and ankle power curves for young (blue) and older (orange) adults during conditions that alter speed (left) and horizontal force (right).

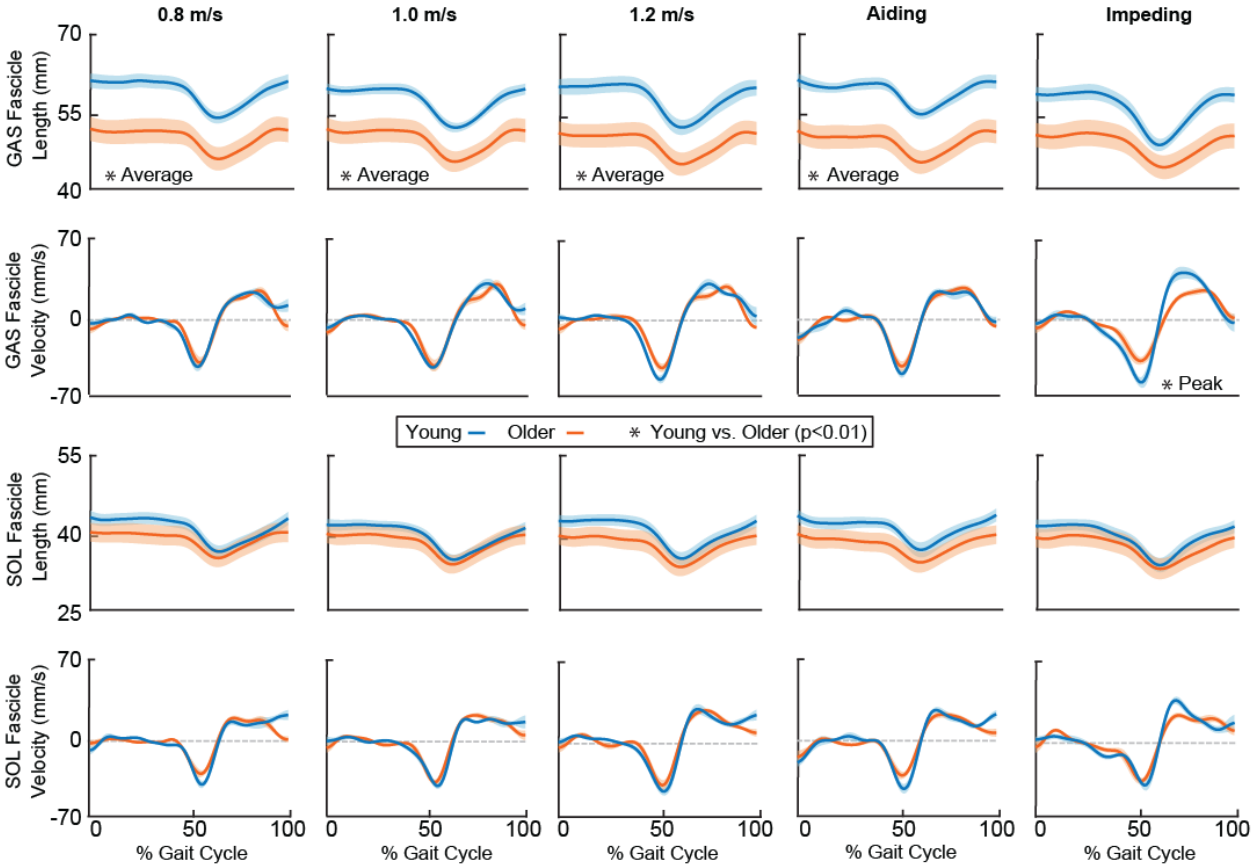

Supplementary Figure S3. Group average (standard error) fascicle length and velocity profiles for the medial gastrocnemius (GAS) and soleus (SOL) muscles. Single asterisks (*) represent significant differences between young (blue) and older (orange) adults (P < 0.01).

Supplementary Table S1. Summary statistics for pairwise comparisons across conditions

	0.8 m/s		1.0 m/s		1.2 m/s ("Normal")		5 %BW Aiding		5 %BW Impeding	
	Young	Older	Young	Older	Young	Older	Young	Older	Young	Older
Plantarflexor Mechanical Output										
Moment (Nm/kg)	1.26±0.19*	1.21±0.10*	1.33±0.15*	1.30±0.12	1.43±0.19	1.36±0.16	1.35±0.12	1.25±0.08	1.50±0.22	1.40±0.14
Power (W/kg)	1.82±0.38*	1.53±0.46*	2.65±0.66*	2.19±0.39*	3.38±0.75	2.74±0.65	2.93±0.78	2.37±0.57	3.61±0.89	2.75±0.67
$^{+}$*Work (J/kg)*	0.21±0.06*	0.18±0.04*	0.28±0.07*	0.24±0.05*	0.33±0.06	0.28±0.05	0.28±0.06	0.24±0.04	0.43±0.09*	0.35±0.06*
Peak Achilles Subtendon Displacement (mm)										
Superficial	3.04±1.18	4.20±0.98*	3.30±1.36	5.10±1.77	**4.01±1.32**	**6.90±1.10**	**2.73±1.09**	**4.78±1.13***	5.12±1.91	7.09±2.91
Deep	5.22±1.36*^	5.34±1.15*^	6.09±1.21^	6.67±1.49*^	7.47±1.50^	8.51±0.91^	5.20±1.46*^	6.28±1.03*^	9.50±1.53*^	8.76±2.67^
Difference	**2.18±0.75***	**1.14±0.37***	**2.79±0.53**	**1.57±0.48**	**3.47±0.77**	**1.61±0.43**	2.47±0.98	1.50±0.52	**4.38±0.81**	**1.67±0.82**
Peak Muscle Length Change (mm)										
MG	6.06±1.69	4.90±1.79	6.48±1.24	5.67±1.91	**8.23±1.15**	**5.53±1.31**	4.82±0.67*	5.02±1.53	**10.35±2.03***	**6.08±1.68**
SOL	5.32±1.43*	4.52±1.41*	5.89±0.93	5.93±1.46	7.17±1.11^	6.15±1.17	4.66±1.30*	4.94±1.33	8.39±1.67^	6.50±2.08
Difference	0.74±1.32	0.38±0.90	0.60±1.18	0.26±1.15	**1.05±0.89**	**0.62±0.57**	0.15±1.25	0.08±1.00	**1.96±0.58***	**0.42±1.12**
Average Fascicle Operating length (mm)										
MG	**59.09±3.74**	**49.98±7.05**	**57.41±2.95**	**49.71±6.52**	**57.91±4.68**	**49.08±6.92**	**59.16±3.33**	**49.19±6.82**	55.99±4.15*	48.63±7.28
SOL	40.91±3.57^	38.77±5.64^	39.51±2.91^	38.06±5.75^	40.29±3.54^	37.65±5.60^	40.99±3.57^	37.74±5.68^	39.21±3.61^	37.29±6.11^
Fascicle Shortening Velocity (mm/s)										
MG	46.42±10.98	44.40±14.74	46.76±5.63	46.63±17.36	58.14±10.49	48.09±8.99	51.14±10.25	48.32±10.26	**64.07±14.89**	**45.62±11.88**
SOL	44.25±7.70*	36.44±9.96	46.50±8.09	42.86±7.48	52.73±10.37	45.25±12.20	47.57±12.16	38.00±6.57^	45.39±14.28^	42.69±11.79

Summary statistics shown are mean±SD, significant age group differences are shown in **bold** (p<0.01), *indicates a within-group speed/force difference compared to walking at 1.2 m/s (p<0.01), ^indicates a within group MG/SOL or superficial/deep difference (p<0.01).

Supplementary Table S2. P-values and effect sizes (Hedges *g*) for Young vs Older adults across conditions

	0.8 m/s		1.0 m/s		1.2 m/s ("Normal")		5 %BW Aiding		5 %BW Impeding	
	p-value (*g*)	*g* 95% CI	p-value (*g*)	*g* 95% CI	p-value (*g*)	*g* 95% CI	p-value (*g*)	*g* 95% CI	p-value (*g*)	*g* 95% CI
Plantarflexor Mechanical Output										
Moment (Nm/kg)	0.41 (0.377)	[-0.54,1.33]	0.71 (0.173)	[-0.74,1.11]	0.40 (0.392)	[-0.52,1.35]	0.07 (0.889)	[-0.04,1.91]	0.27 (0.513)	[-0.40,1.48]
Power (W/kg)	0.17 (0.643)	[-0.27,1.63]	0.09 (0.812)	[-0.11,1.82]	0.07 (0.879)	[-0.05,1.90]	0.10 (0.776)	[-0.15,1.78]	0.03 (1.036)	[0.10,2.08]
$^{+}$*Work (J/kg)*	0.17 (0.639)	[-0.28,1.62]	0.18 (0.627)	[-0.29,1.61]	0.09 (0.817)	[-0.11,1.82]	0.19 (0.612)	[-0.31,1.59]	0.08 (0.848)	[-0.08,1.86]
Peak Achilles Subtendon Displacement (mm)										
Superficial	0.04 (1.020)	[0.08,2.06]	0.03 (1.086)	[0.14,2.14]	**0.00 (2.276)**	**[1.18,3.60]**	**0.00 (1.762)**	**[0.75,2.95]**	0.11 (0.763)	[0.16,1.76]
Deep	0.84 (0.090)	[0.83,1.02]	0.38 (0.406)	[0.51,1.36]	0.10 (0.797)	[0.13,1.80]	0.09 (0.818)	[0.11,1.82]	0.48 (0.324)	[1.27,0.59]
Difference	**0.00 (1.667)**	**[0.66,2.84]**	**0.00 (2.309)**	**[1.21,3.64]**	**0.00 (2.854)**	**[1.65,4.34]**	0.02 (1.183)	[0.23,2.25]	**0.00 (3.173)**	**[1.90,4.76]**
Peak Muscle Length Change (mm)										
MG	0.18 (0.636)	[-0.28,1.62]	0.30 (0.480)	[-0.43,1.44]	**0.00 (2.088)**	**[1.02,3.36]**	0.72 (-0.165)	[-1.10,0.75]	**0.00 (2.183)**	**[1.10,3.48]**
SOL	0.25 (0.535)	[-0.38,1.50]	0.94 (-0.033)	[-0.96,0.89]	0.08 (0.854)	[-0.07,1.87]	0.66 (-0.199)	[-1.14,0.72]	0.05 (0.954)	[0.02,1.98]
Difference	0.50 (0.308)	[-0.61,1.25]	0.14 (0.280)	[-0.63,1.22]	**0.00 (0.558)**	**[-0.36,1.53]**	0.90 (0.059)	[-0.86,0.99]	**0.00 (1.639)**	**[0.64,2.80]**
Average Fascicle Operating length (mm)										
MG	**0.00 (1.537)**	**[0.55,2.68]**	**0.01 (1.449)**	**[0.47,2.57]**	**0.01 (1.423)**	**[0.45,2.54]**	**0.00 (1.770)**	**[0.75,2.96]**	0.02 (1.182)	[0.23,2.25]
SOL	0.35 (0.431)	[-0.48,1.39]	0.51 (0.304)	[-0.61,1.25]	0.25 (0.538)	[-0.38,1.51]	0.17 (0.651)	[-0.27,1.63]	0.43 (0.363)	[-0.55,1.31]
Fascicle Shortening Velocity (mm/s)										
MG	0.75 (0.148)	[-0.77,1.08]	0.98 (0.009)	[-0.91,0.93]	0.04 (0.980)	[0.05,2.01]	0.57 (0.261)	[-0.65,1.20]	**0.01 (1.305)**	**[0.34,2.40]**
SOL	0.08 (0.836)	[-0.09,1.85]	0.34 (0.445)	[-0.47,1.40]	0.18 (0.629)	[-0.29,1.61]	0.05 (0.932)	[0.00,1.96]	0.67 (0.196)	[-0.72,1.13]

Significant age group differences are shown in **bold** (p<0.01).

SUPPLEMENTARY VIDEO CAPTIONS

Supplementary Video V1. Example ultrasound video of the triceps surae muscles during walking (recorded at 76 frames per second, shown at 20% playback speed). An open-source MATLAB routine, UltraTrack[61], quantified time series of medial gastrocnemius and soleus fascicle lengths and pennation angles. Representative fascicles from superficial to deep aponeurosis are shown in yellow.

Supplementary Video V2. Example ultrasound video of the Achilles tendon during walking (recorded at 155 frames per second, shown at 20% playback speed). A 2-D speckle tracking algorithm quantified localized Achilles subtendon tissue displacements from toe off to subsequent toe off[62]. Green dots represent tracked grid of nodes encompassing only Achilles tendon tissue.
