# Supplementary material for: Age-related changes to triceps surae muscle-subtendon interaction dynamics during walking
Source: Sci Rep. 2021 Oct 28;11:21264. doi: 10.1038/s41598-021-00451-y (PMC8553842; doi:10.1038/s41598-021-00451-y)
Supplement: Supplementary file 2 — Supplementary Information 2. [file 41598_2021_451_MOESM2_ESM.pdf]

Supplementary Table S3. Subject Anthropometrics

Subject	Preferred Walking Speed (m/s)	Height (cm)	Gender	Mass (kg)	Age (years)
Young01	1.20	181	M	75.4	22
Young02	1.24	177	M	68.8	21
Young03	1.35	167	F	67.4	24
Young04	1.34	194	M	81.3	20
Young05	1.38	168	F	69.4	23
Young06	1.07	178	F	86.6	20
Young07	1.42	183	M	99.8	27
Young08	1.21	175	M	75.0	30
Young09	1.36	168	F	65.2	30
Older01	1.14	173	M	59.5	77
Older02	1.44	168	M	69.6	75
Older03	1.27	163	F	61.3	69
Older04	0.98	175	M	79.4	71
Older05	1.05	170	F	60.5	77
Older06	1.00	165	M	69.8	78
Older07	1.53	165	M	63.7	67
Older08	1.04	164	F	69.2	77
Older09	1.18	183	M	68.3	75

Supplementary Table S4.
Superficial (GAS) Subtendon Displacement During Stance (mm)

Subject	W08	W10	W12	A12	I12
Young01	-3.3	-4.0	-4.3	-4.3	-8.1
Young02	-2.3	-3.5	-4.4	-2.6	-5.5
Young03	-1.6	-1.9	-3.8	-0.8	-2.4
Young04	-2.1	-3.0	-6.7	-2.8	-7.6
Young05	-3.3	-3.9	-3.8	-3.1	-5.0
Young06	-2.1	-2.1	-2.4	-1.9	-2.6
Young07	-3.7	-2.1	-2.7	-2.4	-5.0
Young08	-3.6	-2.9	-3.0	-2.5	-4.8
Young09	-5.5	-6.3	-5.0	-4.2	-5.1
Older01	-3.8	-6.6	-7.3	-5.5	-7.2
Older02	-4.5	-4.5	-5.7	-3.6	-9.4
Older03	-5.3	-2.6	-7.8	-5.2	-2.6
Older04	-4.1	-3.4	-6.1	-4.1	-7.8
Older05	-5.0	-5.9	-7.3	-6.4	-9.8
Older06	-2.4	-3.5	-7.0	-5.7	-3.9
Older07	-4.7	-8.2	-9.0	-5.1	-11.7
Older08	-4.9	-5.7	-5.6	-4.7	-5.2
Older09	-2.9	-5.5	-6.4	-2.8	-6.2

Supplementary Table S5.
Deep (SOL) Subtendon Displacement During Stance (mm)

Subject	W08	W10	W12	A12	I12
Young01	-5.0	-7.0	-8.7	-6.7	-11.8
Young02	-3.2	-5.5	-7.9	-4.1	-10.1
Young03	-4.3	-4.7	-6.4	-4.2	-7.3
Young04	-4.9	-5.5	-10.2	-5.6	-10.5
Young05	-4.6	-6.4	-6.1	-4.6	-9.6
Young06	-4.7	-4.9	-5.8	-4.1	-7.1
Young07	-6.0	-5.7	-6.0	-3.5	-9.9
Young08	-6.8	-6.5	-7.8	-6.2	-10.4
Young09	-7.6	-8.7	-8.4	-7.8	-8.8
Older01	-4.4	-8.0	-8.9	-7.0	-8.4
Older02	-6.0	-5.8	-7.9	-4.8	-10.6
Older03	-6.7	-5.0	-8.9	-6.2	-5.5
Older04	-5.4	-5.1	-7.8	-5.9	-9.3
Older05	-6.7	-8.0	-9.4	-7.9	-11.1
Older06	-3.5	-5.1	-8.9	-7.5	-4.7
Older07	-5.4	-9.0	-9.9	-6.1	-12.9
Older08	-5.9	-7.0	-7.0	-5.8	-6.9
Older09	-4.0	-7.0	-7.9	-5.4	-9.4

Supplementary Table S6.
Achilles Tendon Non-Uniformity During Stance (mm)

Subject	W08	W10	W12	A12	I12
Young01	1.7	3.0	4.4	2.5	3.7
Young02	0.8	2.0	3.5	1.4	4.6
Young03	2.7	2.9	2.6	3.4	4.9
Young04	2.8	2.5	3.5	2.8	2.9
Young05	1.4	2.4	2.3	1.5	4.6
Young06	2.6	2.7	3.4	2.2	4.5
Young07	2.3	3.5	3.3	1.1	4.9
Young08	3.2	3.6	4.8	3.6	5.6
Young09	2.2	2.4	3.4	3.6	3.7
Older01	0.6	1.4	1.6	1.5	1.2
Older02	1.5	1.3	2.2	1.2	1.2
Older03	1.4	2.4	1.0	1.0	2.9
Older04	1.3	1.7	1.7	1.8	1.6
Older05	1.7	2.2	2.1	1.5	1.3
Older06	1.0	1.6	1.9	1.9	0.7
Older07	0.7	0.8	1.0	1.0	1.2
Older08	1.0	1.3	1.4	1.1	1.7
Older09	1.0	1.5	1.6	2.6	3.2

Supplementary Table S7.
GAS Muscle Length Change During Stance (mm)

Subject	W08	W10	W12	A12	I12
Young01	4.3	6.2	8.8	3.5	12.4
Young02	9.3	6.2	8.7	4.6	13.3
Young03	6.0	5.5	6.5	4.8	7.6
Young04	8.5	8.4	7.5	4.5	8.2
Young05	4.7	6.9	9.1	5.0	9.6
Young06	5.3	6.8	6.9	4.8	9.9
Young07	5.5	4.0	10.1	4.9	12.8
Young08	5.5	7.1	7.7	6.1	9.9
Young09	5.5	7.3	8.7	5.0	9.4
Older01	2.6	4.1	4.3	5.0	5.8
Older02	7.0	7.2	6.4	3.6	6.8
Older03	6.6	3.5	4.3	6.0	4.4
Older04	5.8	7.6	7.3	7.5	6.9
Older05	6.8	8.9	6.9	7.1	9.7
Older06	2.7	4.0	5.7	4.6	3.9
Older07	3.2	4.6	4.3	3.4	6.0
Older08	5.3	4.8	4.0	3.4	5.3
Older09	4.0	6.5	6.5	4.6	5.8

Supplementary Table S8.
SOL Muscle Length Change During Stance (mm)

Subject	W08	W10	W12	A12	I12
Young01	4.1	6.1	6.5	4.7	10.1
Young02	7.5	6.1	6.8	6.1	10.7
Young03	6.0	4.8	6.3	5.2	6.7
Young04	5.5	6.8	6.1	2.6	6.6
Young05	3.3	4.9	7.2	4.5	7.5
Young06	4.0	4.8	7.2	5.2	8.2
Young07	7.0	5.6	9.8	4.3	10.5
Young08	5.8	7.3	7.0	6.5	8.3
Young09	4.5	6.5	7.7	2.9	6.8
Older01	3.4	6.0	5.9	5.3	5.2
Older02	5.9	6.2	6.8	3.3	6.0
Older03	4.7	5.1	5.8	4.8	4.9
Older04	6.3	8.7	8.0	8.0	7.8
Older05	6.1	7.4	7.3	5.0	10.7
Older06	3.1	4.2	5.9	4.7	4.2
Older07	2.3	5.2	4.7	4.7	7.6
Older08	4.5	4.2	4.4	3.6	7.4
Older09	4.4	6.3	6.5	5.0	4.6

Supplementary Table S9.
GAS - SOL Muscle Length Change During Stance (mm)

Subject	W08	W10	W12	A12	I12
Young01	0.1	0.1	2.3	-1.2	2.3
Young02	1.8	0.1	2.0	-1.5	2.6
Young03	-0.1	0.7	0.3	-0.4	0.8
Young04	2.9	1.5	1.4	1.9	1.6
Young05	1.4	2.0	1.9	0.6	2.1
Young06	1.3	2.0	-0.3	-0.4	1.7
Young07	-1.6	-1.6	0.3	0.7	2.3
Young08	-0.2	-0.2	0.7	-0.4	1.5
Young09	1.0	0.8	0.9	2.1	2.6
Older01	-0.8	-1.9	-1.6	-0.4	0.6
Older02	1.0	1.0	-0.4	0.3	0.8
Older03	1.9	-1.6	-1.5	1.2	-0.5
Older04	-0.5	-1.1	-0.7	-0.5	-0.9
Older05	0.7	1.5	-0.5	2.1	-1.0
Older06	-0.4	-0.3	-0.2	-0.1	-0.3
Older07	0.8	-0.7	-0.4	-1.3	-1.6
Older08	0.8	0.5	-0.4	-0.2	-2.1
Older09	-0.3	0.2	0.0	-0.4	1.2

Supplementary Table S10.
Peak Ankle Moment (Nm/kg)

Subject	W08	W10	W12	A12	I12
Young01	1.33	1.35	1.37	1.29	1.43
Young02	1.09	1.27	1.37	1.36	1.47
Young03	1.19	1.36	1.48	1.27	1.48
Young04	1.64	1.53	1.66	1.54	1.92
Young05	1.42	1.42	1.63	1.45	1.63
Young06	1.23	1.35	1.42	1.32	1.48
Young07	1.10	1.16	1.31	1.17	1.35
Young08	1.06	1.05	1.05	1.23	1.10
Young09	1.33	1.49	1.60	1.48	1.63
Older01	1.24	1.41	1.55	1.25	1.37
Older02	1.32	1.37	1.46	1.34	1.56
Older03	1.24	1.35	1.42	1.31	1.42
Older04	1.25	1.26	1.25	1.21	1.29
Older05	1.32	1.48	1.55	1.30	1.64
Older06	1.11	1.17	1.11	1.09	1.25
Older07	1.21	1.39	1.47	1.34	1.50
Older08	1.08	1.14	1.22	1.19	1.28
Older09	1.07	1.17	1.21	1.20	1.27

Supplementary Table S11.
Peak Ankle Power (W/kg)

Subject	W08	W10	W12	A12	I12
Young01	1.76	2.60	3.22	3.11	3.06
Young02	1.61	2.62	3.10	3.53	4.08
Young03	2.09	2.52	3.64	2.68	3.20
Young04	2.06	2.76	3.64	3.09	4.82
Young05	1.88	3.10	3.84	3.14	4.10
Young06	1.06	1.44	2.24	1.91	2.49
Young07	2.13	3.64	4.27	3.15	3.98
Young08	1.52	1.95	2.28	1.61	2.28
Young09	2.26	3.23	4.21	4.17	4.47
Older01	0.66	1.99	2.44	1.89	1.98
Older02	1.97	2.56	3.27	2.54	3.16
Older03	1.90	2.45	3.37	2.98	2.82
Older04	1.73	2.04	2.94	3.09	3.26
Older05	2.02	2.50	3.38	1.90	4.00
Older06	1.28	2.24	1.93	2.49	2.17
Older07	1.50	2.22	2.79	2.73	2.57
Older08	1.69	2.37	2.98	2.43	2.85
Older09	1.05	1.30	1.55	1.32	1.95

Supplementary Table S12.
Positive Ankle Push-Off Work (J/kg)

Subject	W08	W10	W12	A12	I12
Young01	0.20	0.36	0.41	0.34	0.53
Young02	0.16	0.23	0.30	0.32	0.42
Young03	0.17	0.26	0.35	0.24	0.35
Young04	0.30	0.30	0.36	0.34	0.58
Young05	0.22	0.29	0.33	0.26	0.45
Young06	0.16	0.18	0.25	0.15	0.31
Young07	0.26	0.35	0.38	0.26	0.39
Young08	0.14	0.18	0.24	0.25	0.31
Young09	0.26	0.35	0.37	0.34	0.49
Older01	0.12	0.26	0.27	0.32	0.33
Older02	0.22	0.23	0.33	0.22	0.35
Older03	0.20	0.27	0.28	0.26	0.42
Older04	0.16	0.16	0.21	0.21	0.26
Older05	0.18	0.31	0.38	0.21	0.43
Older06	0.10	0.20	0.25	0.30	0.40
Older07	0.23	0.31	0.32	0.25	0.41
Older08	0.19	0.21	0.27	0.24	0.27
Older09	0.19	0.19	0.24	0.19	0.33

Supplementary Table S13.
GAS Average Operating Length (mm)

Subject	W08	W10	W12	A12	I12
Young01	61.2	59.5	55.8	57.0	54.8
Young02	65.8	61.3	65.0	64.1	64.5
Young03	57.8	57.8	58.9	59.9	56.5
Young04	59.6	58.1	59.6	61.3	56.1
Young05	62.3	59.3	63.9	62.6	59.5
Young06	57.8	56.4	57.6	59.5	56.2
Young07	57.9	56.9	54.7	58.7	52.9
Young08	52.5	50.7	49.9	53.6	50.1
Young09	57.0	56.8	55.6	55.7	53.4
Older01	48.7	48.3	46.0	46.9	45.3
Older02	47.5	47.0	46.3	47.6	45.5
Older03	45.4	48.5	47.5	46.7	47.3
Older04	44.8	44.0	43.4	44.0	44.0
Older05	59.8	58.1	57.8	57.6	58.1
Older06	40.9	41.1	40.1	40.4	38.6
Older07	46.0	45.0	45.0	43.7	43.8
Older08	56.5	57.3	55.7	57.2	55.3
Older09	60.2	58.2	59.8	58.6	59.7

Supplementary Table S14.
SOL Average Operating Length (mm)

Subject	W08	W10	W12	A12	I12
Young01	45.4	42.4	42.5	44.5	41.7
Young02	42.2	39.1	35.6	34.4	34.9
Young03	40.3	40.9	41.0	41.8	40.7
Young04	44.1	42.0	45.9	45.5	43.6
Young05	43.0	41.8	41.3	42.6	38.3
Young06	36.0	34.7	41.0	40.4	40.2
Young07	38.5	38.7	40.4	41.6	38.4
Young08	35.5	35.1	34.0	36.4	32.6
Young09	43.2	40.9	40.9	41.7	42.6
Older01	44.7	43.2	42.5	41.9	43.9
Older02	40.2	40.0	38.1	39.1	37.7
Older03	38.3	38.4	38.0	38.6	38.2
Older04	29.0	28.7	29.3	28.9	28.6
Older05	43.1	41.3	42.0	41.9	41.0
Older06	31.9	31.2	30.4	31.0	30.0
Older07	36.2	33.1	32.9	32.1	31.2
Older08	39.8	41.4	40.7	41.2	39.6
Older09	45.7	45.4	44.9	45.0	45.6

Supplementary Table S15.

GAS Peak Shortening Velocity (mm/s)

Subject	W08	W10	W12	A12	I12
Young01	-36.2	-47.7	-62.0	-30.9	-57.1
Young02	-60.5	-48.6	-51.5	-57.7	-88.0
Young03	-48.6	-42.0	-48.7	-39.3	-38.9
Young04	-56.3	-57.7	-55.9	-61.0	-56.1
Young05	-30.7	-42.5	-45.2	-55.9	-84.0
Young06	-33.6	-38.5	-57.2	-49.1	-64.3
Young07	-58.2	-46.0	-78.0	-50.7	-67.9
Young08	-44.6	-51.3	-54.4	-62.2	-57.5
Young09	-49.1	-46.5	-70.4	-53.4	-62.8
Older01	-29.2	-26.3	-49.1	-44.8	-46.4
Older02	-56.3	-48.6	-48.7	-33.3	-51.6
Older03	-61.8	-36.6	-45.4	-54.8	-33.7
Older04	-54.7	-75.2	-61.1	-62.5	-58.9
Older05	-61.9	-73.0	-62.9	-61.1	-66.5
Older06	-29.8	-34.5	-45.0	-34.9	-31.6
Older07	-31.6	-33.2	-34.6	-44.6	-35.1
Older08	-46.2	-40.9	-41.0	-48.4	-46.6
Older09	-28.1	-51.2	-45.2	-50.6	-40.1

Supplementary Table S16.

SOL Peak Shortening Velocity (mm/s)

Subject	W08	W10	W12	A12	I12
Young01	-44.4	-47.7	-44.5	-32.7	-30.1
Young02	-51.4	-49.2	-52.4	-73.6	-62.0
Young03	-52.3	-33.1	-36.5	-45.5	-24.6
Young04	-41.1	-53.9	-53.3	-45.6	-36.1
Young05	-36.4	-41.1	-46.3	-45.5	-64.0
Young06	-32.1	-35.6	-48.3	-49.5	-47.0
Young07	-55.7	-51.3	-64.8	-35.6	-52.9
Young08	-41.6	-56.3	-58.8	-57.5	-35.9
Young09	-43.4	-50.2	-69.7	-42.7	-55.9
Older01	-35.0	-48.5	-58.5	-40.5	-47.9
Older02	-44.8	-44.8	-55.2	-37.2	-40.9
Older03	-40.2	-44.2	-43.7	-31.8	-30.8
Older04	-37.7	-46.5	-46.1	-51.1	-46.2
Older05	-52.8	-51.0	-64.5	-39.9	-68.0
Older06	-24.6	-34.1	-38.5	-27.6	-27.6
Older07	-19.4	-32.4	-25.9	-37.4	-40.4
Older08	-38.4	-33.6	-36.5	-35.4	-46.2
Older09	-34.9	-50.5	-38.5	-41.1	-36.2
